# One size does not fit all. Should gambling loss limits be based on income?

**DOI:** 10.3389/fpsyt.2022.1005172

**Published:** 2022-11-16

**Authors:** Elias Langeland, Ingvild Faxvaag Johnsen, Kaja Kastrup Sømme, Arne Magnus Morken, Eilin Kristine Erevik, Eirin Kolberg, Jakob Jonsson, Rune Aune Mentzoni, Ståle Pallesen

**Affiliations:** ^1^Department of Psychosocial Science, University of Bergen, Bergen, Norway; ^2^Norwegian Competence Center for Gambling and Gaming Research, University of Bergen, Bergen, Norway; ^3^Department of Clinical Neuroscience, Centre for Psychiatry Research, Karolinska Institutet, Stockholm, Sweden

**Keywords:** gambling related harm, PGSI, responsible gambling, precision-recall, receiver operating characteristic, income-specific loss limits, gambling loss limits, low-risk gambling limits

## Abstract

**Background:**

Previous research has suggested empirically based gambling loss limits, with the goal of preventing gambling related harm in the population. However, there is a lack of studies relating gambling loss limits to individual factors such as income. The current study examines whether gambling loss limits should be income-specific.

**Materials and methods:**

The dataset was derived from three representative cross-sectional surveys of the Norwegian population and consisted of 14,630 gamblers. Four income groups, based on a quartile approximation, were formed. Gambling related harm was measured with the Problem Gambling Severity Index (PGSI), and precision-recall (PR) analyses were used to identify loss limits for the different income groups at two levels of gambling severity: moderate-risk gambling and problem gambling.

**Results:**

For both levels of gambling severity, we found the lowest income group to have the lowest gambling loss limits, and the highest income group to have the highest loss limits, which compared to the loss limits for the total sample, were lower and higher, respectively. Calculating the cut-offs for moderate-risk gamblers, we found a consistently ascending pattern from the lowest to the highest income group. Calculating the cut-offs for problem gamblers, we found a similar pattern except for the two middle income groups.

**Conclusion:**

The results suggest that income moderates empirically derived gambling loss limits. Although replication is required, income-based gambling loss limits may have higher applied value for preventing gambling related harm, compared to general loss limits aimed at the entire population.

## Introduction

Gambling is a popular activity in many countries (1) and the availability of gambling seems to be increasing worldwide (2). Online gambling contributes to this availability, and has become increasingly popular in recent years (3, 4). Gambling is, however, an activity that comes at a cost. Problem gambling is present in most countries, with a worldwide prevalence of 0.1–5.8% (1). The fifth edition of the Diagnostic and Statistical Manual of Mental Disorders (DSM-5) includes gambling disorder as a clinical diagnosis comprising nine criteria, of which endorsing four of them within 12 months is sufficient to meet the diagnostic criteria (5). People with gambling disorder experience various forms and levels of gambling related harm, but not everyone who experiences gambling related harm fulfills the criteria for gambling disorder (6). Gambling related harm is a broad term and can for instance refer to negative consequences for one’s economic situation, health, school, and work performance, as well as contribute to criminal activity (7). Furthermore, gambling related harm may affect people around the gamblers, as well as the gamblers themselves (7, 8). Langham et al. (7) suggest the following definition of gambling related harm: “Any initial or exacerbated adverse consequence due to an engagement with gambling that leads to a decrement to the health or wellbeing of an individual, family unit, community or population” (p. 4). As such adverse effects of gambling are experienced across the whole spectrum of gamblers (9), a focus on harm rather than a merely clinical view of gambling issues in prevention efforts is important.

In the context of minimizing gambling related harm, responsible gambling (RG) is often emphasized. Blaszczynski et al. (10) define RG as “policies and practices designed to prevent and reduce potential harms associated with gambling” (p. 308). In order to implement RG, several strategies have been put forward, which are often influenced by two dominant frameworks, the Reno model and the Public Health model. The Reno model has historically had the largest impact. This model emphasizes that gamblers can initiate self-protective behaviors, provided they receive as much information about the gambling product as possible (6). As such, the model emphasizes informed choices, and non-intrusive RG measures targeting mainly risk groups (10–12). However, the Reno model has received criticism for not taking sufficient responsibility for gambling related harm in the entire population (6), and for benefiting gambling companies (11).

The Public Health model is aimed at empirically examining gambling related harm, and social, economic and cultural factors that can cause gambling related harm in the whole population (6, 12, 13). According to this perspective, public health authorities should consider gambling related harm as something everyone can experience, and develop and implement relevant regulatory measures for the entire population (13). Supporters of this view typically welcome state regulations that govern gambling behavior in such a way that gambling related harm decreases for the entire population, e.g., by enforcing mandatory loss- and time limits, and limited access for all, while also targeting vulnerable groups (6, 13).

Norway is one of the few countries worldwide where the state both offers gambling opportunities through a state-regulated monopoly, while also regulating gambling, e.g., through specific legislations and precepts (14). The two Norwegian gambling monopolists, Norsk Tipping and Norsk Rikstoto enforce registered gambling, allowing tracking and regulation of gambling behavior at the individual level. As of today, both companies have an upper loss limit of 20,000 NOK (Norwegian kroner^1^) a month across all games (15, 16). Gamblers can also set personal loss limits, which can be lower (but not higher) than the mandatory limits (17).

Although many people experience harm from gambling, the majority of gamblers do not experience much harm (18–20). This begs the question of what separates the gamblers who experience harm from the gamblers who do not. Research has identified several variables used to conduct binary classifications of gamblers into those experiencing and those not experiencing high levels of gambling related harm. Such classifications have often been based on expenditure, frequency, or duration of gambling. Empirically deriving limits separating gamblers at high risk of harm from gamblers at lower risk levels is a useful tool for developing effective public health policies, such as gambling loss limits.

Research has shown that meeting loss limits related to expenditure and percentage of income perform better than other predictors of gambling related harm (20–22). Louderback et al. (22) found that online gamblers who reached the online loss limit for monthly expenditure were more likely to voluntarily self-exclude or close their account, than those who did not cross the limit. Similarly, in another study, Auer et al. (23) found that very few gamblers used other operators when they reached their loss limits, stating that 71% of “high-risk gamblers” stopped gambling until their loss limit had been reset. This suggests that loss limits can be functional tools for preventing gambling related harm.

Studies in various countries have been conducted to empirically develop gambling loss limits. Currie et al. (24) derived gambling loss limits based on longitudinal data, taken from two independent population cohort studies in Canada. They operationalized harm as having experienced two or more consequences of gambling during the last 12 months, measured by the Problem Gambling Severity Index (PGSI). They found an optimal cut-off at 478 NOK^2^ ($75 CAN) per month, and 1.7% of gross household income (24). Dowling et al. (21) used the same instrument and operationalization of harm as Currie et al. (24). They found a cut-off at 3,291 kr–3,511 NOK ($510–$544 AUD) a year, and 10.2–10.3% of gross personal income. Dowling et al. (20) examined optimal cut-offs in two Australian samples from Tasmania and the Australian Capital Territory. With the same operationalization of harm as the previously mentioned studies, they found a cut-off at 2 452 kr–3,969 NOK ($380–$615 AUD) a year, and 0.83–1.68% of gross personal income. Quilty et al. (25) found a cut-off at 140 NOK ($24.5 CAN) per month using the same operationalization as Currie et al. (24). Half of their Canadian sample was a community sample and the rest a psychiatric outpatient sample. Although there is some variation in these loss limits, they are not dramatically different. Other studies have found quite different loss limits, both higher and lower than the one’s described above. For instance, a study of a German population found that staying below a limit of 260 NOK (€29) per year, strongly reduced the risk of fulfilling any DSM-5 criteria for gambling disorder (26). Louderback et al. (22) found that wagering more than 1,707 NOK (€167.97) a month increased the risk of gambling related harm, while also reporting that losing more than 265 NOK (€26.11) a month increased the risk of gambling related harm. They also reported that spending more than 6.71% of annual household income increases the risk of harm.

Although several studies have been aimed at deriving optimal gambling loss limits, there is a lack of studies investigating if such limits could be more effective by also considering other, non-gambling related variables. Several researchers have suggested that some groups are more at risk for experiencing gambling related harm, and therefore might benefit from more stringent gambling loss limits (22, 24, 26–28). Research suggests that young adults, ethnic minorities, people with low incomes, people previously diagnosed with gambling disorder, and people with substance use disorders, etc., are particularly vulnerable to developing gambling problems (28–31). As some demographic variables are found to be more strongly associated with gambling related harm than others, it is of interest to investigate whether gambling loss limits should be differentiated and adapted to different subgroups of the population, as opposed to being general and absolute. Although not all the mentioned variables are ethically viable for differentiated gambling loss limits, some individual variables, such as income, could be used to develop effective differentiated gambling loss limits.

Studies and population reports reveal that lower socioeconomic status and/or lower income is associated with higher levels of both gambling pathology (31–33) and problem gambling (29). However, there are also studies that do not find an association between lower income and higher levels of gambling related harm or gambling pathology (30, 34–36). Some researchers have addressed income as a variable of interest by deriving gambling loss limits as a percentage of income (20–22, 24). However, to our knowledge, no previous study has estimated specific cut-offs for different income groups. By examining whether gambling loss limits derived from different income groups are substantially different, we can investigate whether gambling loss limits should be adapted to the economic situation of the gambler. Specific loss limits for specific income groups may also be easier to implement as public health policies than loss limits as a percentage of income.

Accordingly, the purpose of the current study was to investigate if gambling loss limits could be improved by being based on gamblers’ income. To do so, we aimed to derive optimal gambling loss limits for four different income groups, based on data collected from three independent Norwegian population-based studies (29, 37, 38). It should be noted that the concept of loss limits often reflect loss limits set by the customers themselves (39). However, in the present study the concept of loss limits refers to limits derived from binary data-based classification analyses. Another aim of the study was to identify and compare optimal cut-offs when setting the threshold of classification at moderate-risk gamblers (PGSI-score of three or more) and problem gamblers (PGSI-score of eight or more). We wanted to investigate if the same trend emerged for both classifications. Loss limits derived by setting the threshold of classification at moderate-risk gamblers seems to correspond well with the concept of low-risk gambling limits, a term used in several previous studies (20, 21, 24). Our hypotheses were: (1) estimated cut-offs increase with income group (i.e., are lower for lower income groups and higher for higher income groups); and (2) cut-offs are lower, but show the same ascending pattern, when setting the threshold of classification at moderate-risk gamblers than when setting the threshold at problem gamblers.

## Materials and methods

### Sample

The dataset consisted of data collected from three cross-sectional population surveys examining the extent of gambling problems in Norway. The surveys were conducted in 2013, 2015, and 2019, and funded by the Norwegian Gambling Authority. Participants (ages 16–74 years) were randomly selected from the National Population Registry of Norway, and answered the questionnaire electronically or in paper format. The questionnaires consisted by and large of the same items in all three surveys. The response rate was 43.6% in 2013 (*N* = 10,081), 40.8% in 2015 (*N* = 5,485) and 32.7% in 2019 (*N* = 9,248), respectively. The surveys conducted in 2013 and 2015 were approved by the Regional Committee for Medical and Health Research Ethics for Health Region West (2013/120/REK vest), and the survey conducted in 2019 was approved by the Norwegian Center for Research Data (No. 528056). A more detailed description of the samples and procedure can be found in the original sources (29, 37, 38).

Only respondents who had participated in gambling during the last 12 months and who had answered the questions relevant for the current analyses were included in the current sample. Variables of interest in the current study were annual income, expenditure on gambling during the last 12 months, and scores on the PGSI. Data from respondents who answered that they had gambled, but did not state that they had spent money on any of the different gambling activities listed in the questionnaire, were also excluded. Missing answers on the harm measure (PGSI) were, following a conservative approach, replaced by “0.” The final analytic sample comprised 14,630 participants (59% of the original samples). Men accounted for 51.7% of the sample, and ages ranged from 16 to 74 years (*M* = 47.8; SD = 15.3). Problem gamblers made up 1.2% of the sample, while moderate-risk gamblers made up 3.7%. Further details regarding demographics are presented in Table 1.

**TABLE 1:** Demographic characteristics of the sample.

Variable	*n* (%)
**Gender**	
Men	7,566 (51.7)
Women	7,064 (48.3)
**PGSI-category**	
Problem gamblers (PGSI ≥ 8)	176 (1.2)
Moderate-risk gamblers (7 ≥ PGSI ≥ 3)	547 (3.7)
Low-risk gamblers (2 ≥ PGSI ≥ 1)	1,869 (12.8)
Non-problem gamblers (PGSI = 0)	12,038 (82.3)
**Level of education**	
Not completed primary school	41 (0.3)
Primary school (grade 1–10)	1,202 (8.2)
High school (grade 11–13)	3,401 (23.2)
Vocational training	3,303 (22.6)
University/college (up to 4 years)	4,356 (29.8)
University/college (5–6 years)	2,171 (14.8)
Ph.D.	123 (0.8)
Missing data	33 (0.2)
**Occupational status**	
Full-time employee	8,271 (56.5)
Part-time employee	1,446 (9.9)
Student	1,016 (6.9)
Stay-at-home/retired	2,186 (14.9)
Unemployed/disabled/vocational rehabilitation/work assessment allowance	1,550 (10.6)
Missing data	161 (1.1)
**Country of birth**	
Norway	13,179 (90.1)
Nordic country outside Norway	344 (2.4)
European country outside the Nordic countries	450 (3.1)
Africa	66 (0.5)
Asia	231 (1.6)
North America	53 (0.4)
South or Middle America	45 (0.3)
Oceania	5 (<0.01)
Missing data	257 (1.8)
**Children living in home**	
None	9,328 (63.8)
1 child	2,051 (14.0)
2 children	2,301 (15.7)
3 children	723 (4.9)
4 children	97 (0.7)
5 children or more	42 (0.3)
Missing data	88 (0.6)
**Marital status**	
Cohabitant/married	10,221 (71.4)
Single/separated/divorced/widow/widower	4,064 (27.8)
Missing data	125 (0.9)

*N* = 14,630. PGSI, problem gambling severity index. Participants were on average 47.8 years old (SD = 15.3) and ages ranged from 16 to 74.

### Measurements

#### Annual income and income groups

In the questionnaire, income was defined as “personal income before tax the last year”. The item had 11 response alternatives, each with a range of 100,000 NOK (starting with “0 NOK–99,999 NOK”), except the highest category which was “1,000,000 NOK or more” (29, 37, 38). The 11 categories were (based on quartiles) collapsed into four income groups by using the most even distribution of the income categories. The range for income group one (IG-1) was 0 NOK–199,999 NOK in all three surveys. For income group two (IG-2) the range was 200,000 NOK–399,999 NOK in all three surveys. For income group three (IG-3) the range was 400,000 NOK–499,999 NOK in 2013 and 2015, and 400,000 NOK–599,999 NOK in 2019. For income group four (IG-4) the range was 500,000 NOK and more in 2013 and 2015, and 600,000 NOK and more in 2019. Out of the total sample (*N* = 14,630), IG-1 consisted of 2,190 participants (15%), IG-2 consisted of 4,273 participants (29.2%), IG-3 consisted of 3,749 participants (25.6%) and IG-4 consisted of 4,418 participants (30.2%).

#### Annual expenditure on gambling

To assess annual expenditure on gambling, the following question was asked: “Check off for approximately how much money (in NOK) you have spent on gambling during the last 12 months in the following games:”, e.g., scratch cards, sports betting, horse race betting, internet-based casino games, internet-based poker, and lotteries. The gambling expenditure on each game was assessed using the following ranges: “Nothing/not gambled,” “1 NOK–1,000 NOK,” “1,001 NOK–5,000 NOK,” “5,001 NOK–10,000 NOK,” “10,001 NOK–25,000 NOK,” and “more than 25,000 NOK.” To be able to calculate proxy for gambling expenditure, the midpoints of the intervals were used in the calculations. For the highest category (“more than 25,000 NOK”) the value was set to 30,000 NOK. A composite score for gambling expenditure was then calculated by adding the expenditure for all games listed in the questionnaire. To adjust for the inflation over the years, expenditures were converted to the 2021-value of the NOK.

#### Problem gambling severity index

Level of experienced gambling related harm was measured using the PGSI, which is a subscale of the more extensive Canadian Problem Gambling Index (CPGI) (40), that has been developed for use in general population surveys (41). The PGSI consists of nine items that produce a composite score reflecting an individual’s level of problem gambling (41). The PGSI has previously been found to have adequate psychometric properties with a Cronbach’s alpha of 0.86 (42). In the present study, the Cronbach’s alpha was acceptable, with a value of 0.89.

Five of the PGSI-items address gambling behavior, for instance: “Have you bet more than you could really afford to lose?”, and four items address consequences, for instance “Has your gambling caused any financial problems for you or your household?” (41). The responses are provided along a four-point scale ranging from 0 (“never”), one (“sometimes”), two (“most of the time”), to three (“almost always”) (41). Accordingly, the composite score for each participant is between 0 and 27. Based on the composite score, the gamblers are categorized into one of four categories: non-problem gambler (with a score of 0), low-risk gambler (with scores of 1–2), moderate-risk gambler (with scores of 3–7), and problem gambler (with scores of 8–27) (41).

For the present analyses, we made binary classifications of the participants based on their PGSI-score. The first classification (hereby called the moderate-risk gambler analyses) separated gamblers with a PGSI-score of three or higher (moderate-risk gamblers and problem gamblers) from gamblers with a PGSI-score of two or lower. For the second set of analyses (hereby called the problem gambler analyses) the classification separated gamblers with a PGSI-score of eight or higher (problem gamblers) from gamblers with a PGSI-score of seven or lower. In other words, in the moderate-risk gambler analyses, both moderate-risk gamblers and problem gamblers were defined as positive cases, and in the problem gambler analyses, only problem gamblers were defined as positive cases. The number of moderate-risk gamblers and problem gamblers in the current sample is shown in Table 1.

### Statistical analysis

Precision-recall (PR) analyses were performed to identify the optimal cut-offs for gambling expenditure classifying positive cases and negative cases. This was done for the total sample and the four income groups, for both levels of classification. That is, in the moderate-risk gambler analyses, positive cases were those classified as moderate-risk gamblers or problem gamblers, and in the problem gambler analyses, positive cases were only those classified as problem gamblers. Another common analysis for classifier performance over a range of tradeoffs between true positive and false positive rates, is the receiver operating characteristic (ROC) analysis (43). Using ROC analysis, a useful predictive model must have an area under the curve (AUC) that is higher than 0.50 (44), which would be a random classifier. In ROC analysis, the Youden Index is commonly used for determining optimal cut-offs, combining sensitivity (the true positive rate) and specificity (the true negative rate) into a single measure (45, 46). However, for heavily imbalanced datasets, ROC analyses are often misleading (47, 48). PR analyses are better suited in cases with imbalanced datasets. Since the positive rates in our income groups ranged from 0.5% to 10.4% (making it imbalanced), we conducted PR analyses to derive cut-offs. Similarly to ROC analysis, PR analyses also require a dichotomous outcome variable (47), classifying participants as either positive or negative cases. PR analyses provide PR curves showing precision ($\frac{t\,r\,u\,e\,p\,o\,s\,i\,t\,i\,v\,e}{t\,r\,u\,e\,p\,o\,s\,i\,t\,i\,v\,e+f\,a\,l\,s\,e\,p\,o\,s\,i\,t\,i\,v\,e}$) and recall ($\frac{t\,r\,u\,e\,p\,o\,s\,i\,t\,i\,v\,e}{t\,r\,u\,e\,p\,o\,s\,i\,t\,i\,v\,e+f\,a\,l\,s\,e\,n\,e\,g\,a\,t\,i\,v\,e}$) for each point on the curve, in the present case corresponding to annual gambling expenditure. In contrast to ROC analyses, where the AUC of a good model needs to exceed 0.50, the AUC for a useful model using PR analysis needs to exceed their baseline model, which is equal to the proportion of positive cases (48). A common indicator for optimal cut-offs when using PR analysis is the F1-score ($2\times \frac{p\,r\,e\,c\,i\,s\,i\,o\,n\times r\,e\,c\,a\,l\,l}{p\,r\,e\,c\,i\,s\,i\,o\,n+r\,e\,c\,a\,l\,l}$), which is based on a tradeoff between precision and recall. An F1-score is the harmonic mean of precision and recall for a given point (49). In order to demonstrate the difference in results between ROC- and PR analyses for an imbalanced dataset, we also conducted a ROC analysis for the total sample, setting the threshold of classification at moderate-risk gamblers. The results from the ROC analysis, with a cut-off based on the Youden Index, were compared to the results from the PR analysis, with a cut-off based on the F1-score. The analyses were conducted using IBM SPSS version 28, and the PRROC package (50) for R.

## Results

The cut-offs derived from our PR analyses are shown in Table 2 (moderate-risk gambler analyses) and Table 3 (problem gambler analyses) together with corresponding precision-, recall-, and F1-scores. The tables also present the number of participants and the percentage of positive cases in each income group. The area under the precision-recall curve (PR-AUC), and corresponding baseline model, comprising the PR-AUC of a random classifier (48), is also shown for each analysis, for comparison purposes.

**TABLE 2:** Results from the precision-recall (PR) analyses when setting the threshold of classification at moderate-risk gamblers.

Group	Total no. in group	Positive cases^a^ (%^b^)	Precision	Recall	F1-score	Cut-off (NOK per year)	Cut-off (NOK per month)	PR-AUC	Baseline model
Total sample	14,630	723 (4.9)	0.53700	0.24100	0.33269	23,033.96	1,919.50	0.3001301	0.049
IG-1	2,190	227 (10.4)	0.36100	0.53300	0.43045	4,683.39	390.28	0.4193924	0.104
IG-2	4,273	234 (5.5)	0.34800	0.30800	0.32678	13,777.74	1,148.15	0.3094834	0.055
IG-3	3,749	141 (3.8)	0.68300	0.30500	0.42169	26,420.88	2,201.74	0.3836997	0.038
IG-4	4,418	121 (2.7)	0.44200	0.31400	0.36716	27,820.82	2,318.40	0.2648659	0.027

PR, precision-recall; IG, income group; NOK, Norwegian kroner; PR-AUC, area under the precision-recall curve. ^a^Positive cases = Number of moderate-risk gamblers and problem gamblers. ^b^% = Percent of positive cases in the group.

**TABLE 3:** Results from the precision-recall (PR) analyses when setting the threshold of classification at problem gamblers.

Group	Total no. in group	Positive cases^a^ (%^b^)	Precision	Recall	F1-score	Cut-off (NOK per year)	Cut-off (NOK per month)	PR-AUC	Baseline model
Total sample	14,630	176 (1.2)	0.45600	0.35200	0.39731	41,923.53	3,493.63	0.2737431	0.012
IG-1	2,190	57 (2.6)	0.57500	0.40400	0.47457	23,299.86	1,941.66	0.4052243	0.026
IG-2	4,273	59 (1.4)	0.5500	0.37300	0.44453	41,923.53	3,493.63	0.3054401	0.014
IG-3	3,749	38 (1.0)	0.41700	0.52600	0.46520	31,963.14	2,663.60	0.3728835	0.010
IG-4	4,418	22 (0.5)	0.22200	0.36400	0.27580	45,585.06	3,798.76	0.1572953	0.005

PR, precision-recall; IG, income group; NOK, Norwegian kroner; PR-AUC, area under the precision-recall curve. ^a^Positive cases = Number of problem gamblers. ^b^% = Percent of positive cases in the group.

The cut-offs from the moderate-risk gambler analyses were consistently ascending, with higher income groups having higher cut-offs. The cut-off for the total sample was higher than the cut-offs for IG-1 and IG-2, and lower than the cut-offs for IG-3 and IG-4. All cut-offs from the moderate-risk gambler analyses were lower than the corresponding cut-offs from the problem gambler analyses (see Tables 2, 3).

The cut-offs from the problem gambler analyses were generally ascending, with higher cut-offs for income groups with a higher income, except for IG-2. This income group had a somewhat higher cut-off than IG-3, though still being lower than the cut-off for IG-4. The cut-off for the total sample was higher than the cut-offs for IG-1 and IG-3, lower than the cut-off for IG-4, and the exact same as the cut-off for IG-2 (see Table 3).

All PR curves showed an AUC that was considerably higher than their respective baseline model (see Tables 2, 3), being 4.0–37.3 times higher than their baseline models. The PR curves in the moderate-risk gambler analyses (see Supplementary Figure 1) had AUCs being 4.0–10.1 times higher than their baseline model. For PR curves in the problem gambler analyses (see Supplementary Figure 2), AUCs were 15.6–37.3 times higher than their baseline model. The ratios between AUC and baseline were thus consistently higher for PR curves for the problem gambler analyses than for PR curves for the moderate-risk gambler analyses.

When conducting a ROC analysis for the total sample, distinguishing moderate-risk gamblers and problem gamblers from the rest, we found an optimal cut-off at 4,243 NOK per year (which equals 354 NOK per month). The corresponding cut-off from the PR analysis for the total sample was 23,034 NOK per year (which equals 1,920 NOK per month). This is approximately 5.4 times higher than the cut-off derived from the ROC analysis. The ROC curve (see Supplementary Figure 3) had an AUC of 0.75. Details concerning the ROC analysis (sensitivity, specificity, Youden Index, ROC-AUC, etc.) can be found in Table 4, which also shows the results from the PR analysis in the moderate-risk gambler analysis for the total sample, for comparison. Sensitivity, specificity, and Youden Index are not shown for the PR analysis in Table 4, because these values are not used when conducting PR-analyses.

**TABLE 4:** Results from the receiver operating characteristic (ROC) analysis and the precision-recall (PR) analysis when setting the threshold of classification at moderate-risk gamblers.

Analysis	Group	*N*	Positive cases^a^ (%^b^)	Sensitivity	Specificity	Youden index	Cut-off (NOK per year)	Cut-off (NOK per month)	AUC	Baseline model
ROC analysis	Total sample	14,630	723 (4.9)	0.62700	0.77700	0.40400	4,242.55	353.55	0.748	0.50
PR analysis	Total sample	14,630	723 (4.9)				23,033.96	1,919.50	0.3001301	0.049

ROC, receiver operating characteristic; PR, precision-recall; NOK, Norwegian kroner; AUC, area under the curve. ^a^Positive cases = number of moderate-risk gamblers and problem gamblers. ^b^% = percent of positive cases in the sample.

## Discussion

The main purpose of the current study was to investigate if gambling loss limits could be improved by being based on gamblers’ income. In line with our first hypothesis, optimal cut-offs were in most cases lower for lower income groups. One exception was IG-2 in the problem gambler analyses, for which we found a somewhat higher cut-off than IG-3. In line with our second hypothesis, the results showed that optimal cut-offs were lower in all the moderate-risk gambler analyses, compared to the corresponding problem gambler analyses. These results thus generally support our hypothesis that estimated cut-offs are lower for lower income groups and higher for higher income groups. This was especially true for experiencing moderate-risk harm, where the pattern of cut-offs was consistently ascending from the lowest to the highest income group. The cut-offs related to problem gamblers did not show the same consistent pattern. This may suggest that other factors than income play an important role in experiencing gambling related harm, especially at higher levels of harm. Still, the cut-offs from the problem gambler analyses were lowest for the lowest income group, and highest for the highest income group, which provides partial support for our first hypothesis. In support of our expectations, every PR curve had an AUC that was considerably higher than their respective baseline models, implying that gambling expenditure predicts moderate-risk gambling and problem gambling beyond random classification. This supports the validity of the cut-offs derived through PR analysis using gambling expenditure as a classifier.

The value of determining cut-offs based on different income groups is substantiated especially when comparing the cut-offs derived from the lowest income group to the cut-offs derived from the total sample. For instance, the cut-off for IG-1 in the moderate-risk analysis was 4,683 NOK per year, while the cut-off for the total sample was 23,034 NOK per year. This suggests that people in lower income groups might experience gambling related harm by following the gambling loss limits derived from the total sample. In contrast, the cut-off in the moderate-risk analysis for IG-4 was 27,821 NOK per year, which is slightly higher than the cut-off for the total sample, which was 23,034 NOK per year. This suggests that people in the highest income group may exceed the gambling loss limits derived from the total sample with a lower probability of being moderate-risk- or problem gamblers. A similar pattern was found in the problem gambler analyses (see Table 3). These results support that individualized cut-offs taking personal income into account is especially important for people with a low income. The present study shows a higher prevalence of gambling related harm among lower income groups than higher income groups (see Tables 2, 3). These prevalence rates are in line with previous studies showing that people with a low socio-economic status may be more predisposed to gambling related harm (31, 33), substantiating the need for income-specific loss limits.

The results show, in line with the second hypothesis, that there was a considerable difference in cut-offs based on severity of gambling related harm. Setting the threshold of classification at moderate-risk gamblers resulted in a cut-off at 23,034 NOK per year for the total sample. When setting the threshold of classification at problem gamblers, we found a cut-off at 41,924 NOK per year. The magnitude of this difference was even larger for IG-1, where the cut-off for the moderate-risk analysis was 4,683 NOK per year, while the cut-off for the problem gambler analysis was 23,300 NOK per year. This suggests that the operationalization of gambling related harm has a large impact on derived gambling loss limits.

Compared to previous studies on gambling loss limits, the cut-offs for the total sample from the present study were generally higher. For instance, Currie et al. (24) found a low-risk limit at 478 NOK per month, Dowling et al. (21) found low-risk limits at 274 NOK–293 NOK per month, and Dowling et al. (20) found low-risk limits at 204 NOK–331 NOK per month. The operationalizations of harm in these studies are comparable to the moderate-risk gambler analysis for the total sample in the present study. Our result from this analysis was 1,920 NOK per month which is clearly higher. However, when deriving cut-offs for different income groups, the present study found a cut-off at 390 NOK per month for the lowest income group in the moderate-risk gambler analysis. This cut-off is closer to those reported by the aforementioned studies. A study which found a more similar cut-off to the moderate-risk gambler analysis of the total sample in the present study was that of Louderback et al. (22). They reported that gambling for more than 1,707 NOK per month increased the risk of gambling related harm. It should be noted that Louderback et al. (22) used actual tracking data and a different harm measure (the Brief Biosocial Gambling Screen) than the aforementioned studies and the present study, which all relied on the PGSI.

There may be several reasons for the differing findings between the present study and previous studies. First, the operationalization of harm has varied across studies, which makes direct comparison difficult and imprecise. In line with this, our study found differing cut-offs for the total sample when setting the threshold of classification at moderate-risk gamblers and problem gamblers; the cut-off from the problem gambler analysis being 1.8 times as high. This underlines how different operationalizations of harm have a large impact on empirical gambling loss limits. Socioeconomic differences between countries and differences in data collection may also play a role in terms of the differing findings. In addition, our study is the only one to have used PR analyses instead of ROC analyses to derive cut-offs.

Conducting a ROC analysis for the total sample with a threshold of classification at moderate-risk gamblers resulted in a cut-off at 354 NOK per month (4,243 NOK per year), as opposed to using PR analysis, which resulted in a cut-off at 1,920 NOK per month (23,034 NOK per year). Although seemingly quite different, both the PR analysis and the ROC analysis had AUCs well above their respective baseline models (see Tables 2, 4). When comparing the different cut-offs, it is apparent that ROC and PR analyses are not interchangeable. As our sample was heavily imbalanced, using a ROC analysis is deemed inappropriate (47, 48). The cut-off from the present ROC analysis thus has no value beyond the purpose of comparing it to cut-offs from previous studies and the cut-off from the current PR analysis. Still, most previous attempts at finding monetary cut-offs for gambling have been made using ROC analyses. Most of these studies have found cut-offs that are considerably lower than the ones derived from the PR analyses in the present study. This can, as previously described, be attributed to several factors, but it is worth pointing out that the results from our ROC analysis more closely resembles cut-offs from previous studies [e.g., (20, 21, 24, 25)]. As most gamblers are non-problem gamblers or low-risk gamblers (18–20), most studies using a representative sample of gamblers will have imbalanced samples, making PR analysis a more appropriate approach (47, 48). The magnitude of the difference between cut-offs derived using PR- and ROC analysis should be more thoroughly investigated in future studies. We only reported one such example, as further illustrations of this issue are beyond the scope of the present study.

### Limitations and strengths

Questions regarding both annual income and annual gambling expenditure were measured using intervals in terms of NOK, and expenditure was estimated based on the midpoint of the interval. For the highest expenditure interval (“more than 25,000 NOK”), a value of 30,000 NOK was set. These estimates may thus be inaccurate, and they should as such be regarded as proxies for the actual figures. Another limitation relates to the fact that the sample sizes of the income groups differed somewhat, and that the ranges of IG-3 and IG-4 were not the same across all of the original surveys. As a result, there is some inconsistency between the members of IG-3, as well as IG-4 in the present study. Further, the variables were measured using self-report, and studies show that online gambling expenditure are reported inaccurately when using self-report compared to objective tracking data (51, 52). In addition, the questionnaire was formulated as “money spent on gambling” [for original Norwegian wording, see Pallesen et al. (29, 37, 38)]. This can potentially be interpreted as either money lost on gambling, or money wagered, hence some ambiguity seems to be present in terms of how this item was formulated. Other researchers have differentiated between the two, for instance Louderback et al. (22) who measured both “amount wagered per month” and “net outcome of gambling per month.” In that study, they found quite different cut-offs for these two measures. This illustrates the importance of differentiating between the two constructs. In addition, the present study examined annual income, while other factors related to one’s economy, as fortune and debt, was not considered. These factors could potentially have an impact on how much money a person can spend on gambling without experiencing higher levels of harm.

The PGSI, which was used for assessing harm in the present study, has met criticism for emphasizing financial harm more than other types of harm (21, 27). Research has identified several non-financial domains of harm (7), and the PGSI may lack sensitivity by emphasizing one domain more than others. Considering that the present study investigated the predictive value of money spent gambling, the relationship between this variable and gambling related harm may be inflated due to the PGSI’s emphasis on financial harm. Investigating the relationship between gambling expenditure and other domains of gambling related harm could thus be an interesting prospect for future studies. Still, the PGSI is a widely used measure of gambling related harm, and the use of this measure makes comparison to other studies more valid.

Another limitation concerns the replacement of missing values on the PGSI with the value “0” which was conducted in order to avoid overestimation of gambling problems. However, only 0.83% of the respondents had missing data and only about 0.20% of all the responses were missing. Due to the left-skewed distribution of the scores on the PGSI and the few missing data points, the missing data replacement procedure used in the present study had likely negligible influence on the results compared to more formal imputation techniques.

Considering that the present study was cross-sectional, the findings do not allow conclusions of directionality or causality. In other words, exceeding the loss limits derived in the current study may not cause gambling related harm. Similarly, staying below the limits does not necessarily prevent experiencing gambling related harm. To draw such conclusions, longitudinal studies would be required. The current loss limits predict whether a gambler experiences gambling related harm based on concurrent reporting of gambling expenditure, hence the limits do not predict whether or not the gambler will experience gambling related harm in the future. In addition, other confounding variables (e.g., fortune, debt, and childcare responsibility) may have an impact on the relationship between gambling expenditure and gambling related harm. Regardless of the limitations, the present study has several strengths, and provides novel information to the field of gambling loss limits. A strength of the current study is the large and representative sample, consisting of 14,630 gamblers, recruited based on random sampling from the National Population Registry of Norway. Furthermore, the present study is the first study deriving gambling loss limits across different income groups. This may be of great applied value, providing more responsible and individualized gambling loss limits. In addition, this is the first study to use PR analysis to derive empirical gambling loss limits. Using ROC analysis for imbalanced datasets may exaggerate the accuracy of the classifier by emphasizing true negatives (53). This is prevented by using PR analysis, as neither precision nor recall is affected by true negatives. As illustrated above, ROC analysis may also result in vastly different cut-offs compared to those derived by PR analysis, highlighting the importance of applying adequate statistical analyses. In addition, the present study derived cut-offs from two different thresholds of classification, both at moderate-risk gamblers and problem gamblers. This provides information regarding different risk levels, and may thus be relevant for gamblers experiencing different levels of harm.

### Implications

By being income-specific, the gambling loss limits derived in the present study may have great applied value. From a public health perspective, one might argue that a conservative approach is favorable to prevent gambling related harm in the population. On the other hand, such an approach could interfere with the recreational side of gambling, as emphasized in the Reno model. Global loss limits may ensure some protection and recreation for the population as a whole, but may not be sufficiently conservative for lower income groups, and may be too conservative for higher income groups. Income-specific loss limits, on the other hand, may ensure both protection and recreation across income groups.

The findings in the present study can also contribute to the further development of quantitative guidelines for responsible gambling. Research on gambling loss limits in countries such as Canada has helped develop guidelines that apply to the entire population (54). Income-specific loss limits are relevant to the development of such national guidelines and may also be used by gambling operators. They could also be beneficial by enabling consumers to make informed choices about personal risk, such as setting personal loss limits that are appropriate to their income. In addition, income-specific mandatory loss limits could also be implemented in the form of affordability checks (55, 56). Furthermore, income-specific cut-offs can serve as a simple and cost-effective way for gambling operators to identify and flag potential moderate-risk- and problem gamblers (20).

Income-specific loss limits can also generate public discussion about the current global loss limits enforced by the Norwegian monopolists, Norsk Tipping and Norsk Rikstoto. The cut-offs derived from the current study are considerably more conservative than these limits, which are global monthly loss limits of 20,000 NOK (15, 16). In the current study, even the cut-off derived from the highest income group in the problem gambler analysis was less than a fifth of the upper global loss limits set by Norsk Tipping and Norsk Rikstoto. Findings from the present study, as well as loss limits found in other studies, thus indicate that the loss limits set by Norsk Tipping and Norsk Rikstoto are quite high, and therefore may not be able to prevent gamblers from experiencing considerable gambling related harm. Still, it should be noted that both monopolists encourage their customers to set individual loss limits that may be far lower than the mandatory limits.

Despite the many possible implementations and benefits of gambling loss limits, it is important to emphasize that staying below loss limits should not contribute to an illusory sense of safety. Gamblers staying below loss limits can experience gambling related harm. Highlighting this, a study in New Zealand by Browne et al. (9), found that 48% of gambling related harm could be attributed to low-risk gambling. In other words, harm is not only experienced by moderate-risk- and problem gamblers or people exceeding gambling loss limits. In line with this, there is some debate regarding the value and interpretation of gambling loss limits as a functional tool. Currie et al. (24, 57) reported that gambling risk curves are J-shaped. This provides support for the applicability of gambling loss limits, as staying below the suggested limits would be associated with low risk of harm and exceeding the limits would mean a sharp increase in risk. However, other studies (58, 59), have found gambling risk curves to be linear or r-shaped, contradicting the sudden increase in risk of harm with increasing losses. These findings underline that the thresholds should be considered thresholds of acceptable risk, rather than thresholds of safe gambling. To better understand how gambling loss limits can be understood and applied, further research is required regarding the shape of gambling risk-curves.

### Directions for future research

Being the first study using PR analysis and income groups to derive gambling loss limits, replication is required. Specific income groups and PR analysis could also be used to examine factors not investigated in the present study. Such factors include specific cut-offs for specific games, cut-offs for time spent gambling per session, and cut-offs for times gambled per month or year. Previous studies have investigated such factors (24, 25, 60), but not by using different income groups or PR analysis. Furthermore, studies including other economic factors (such as fortune, debt, and childcare responsibility), may also add predictive value beyond information about income only. More studies based on actual tracking data, in contrast to self-report, could also be conducted to investigate the validity of the present- and previous findings. In addition, most studies on gambling loss limits have been cross-sectional. To draw conclusions regarding directionality, more longitudinal studies should be conducted, and these could also benefit from a PR analysis approach and investigating cut-offs for different income groups. Further studies using this approach should also be conducted in other countries to investigate the generalizability of the current findings.

## Data availability statement

The raw data supporting the conclusions of this article will be made available by the authors, without undue reservation.

## Ethics statement

The studies involving human participants were reviewed and approved by Regional Committee for Medical and Health Research Ethics for Health Region West (2013/120/REK vest) and Norwegian Center for Research Data (No. 528056). Written informed consent from the participants’ legal guardian/next of kin was not required to participate in this study in accordance with the national legislation and the institutional requirements.

## Author contributions

EL, IJ, and KS wrote the first draft of the manuscript and conducted the analyses in SPSS. SP designed the study and contributed to subsequent drafts of the manuscript. EK conducted the analyses in R. AM, EE, EK, JJ, RM, and SP critically revised the manuscript. All authors contributed to the article and approved the submitted version.

## Footnotes

1 €1 ≈ 10 NOK.

2 Currencies from previous studies are converted to Norwegian kroner by the mean exchange rate of the publication year of the studies.
