# Supplementary material for: One size does not fit all. Should gambling loss limits be based on income?
Source: Front Psychiatry. 2022 Nov 16;13:1005172. doi: 10.3389/fpsyt.2022.1005172 (PMC9709812; doi:10.3389/fpsyt.2022.1005172)
Supplement: Supplementary file 1 [file Data_Sheet_1.DOCX]

Supplementary Material


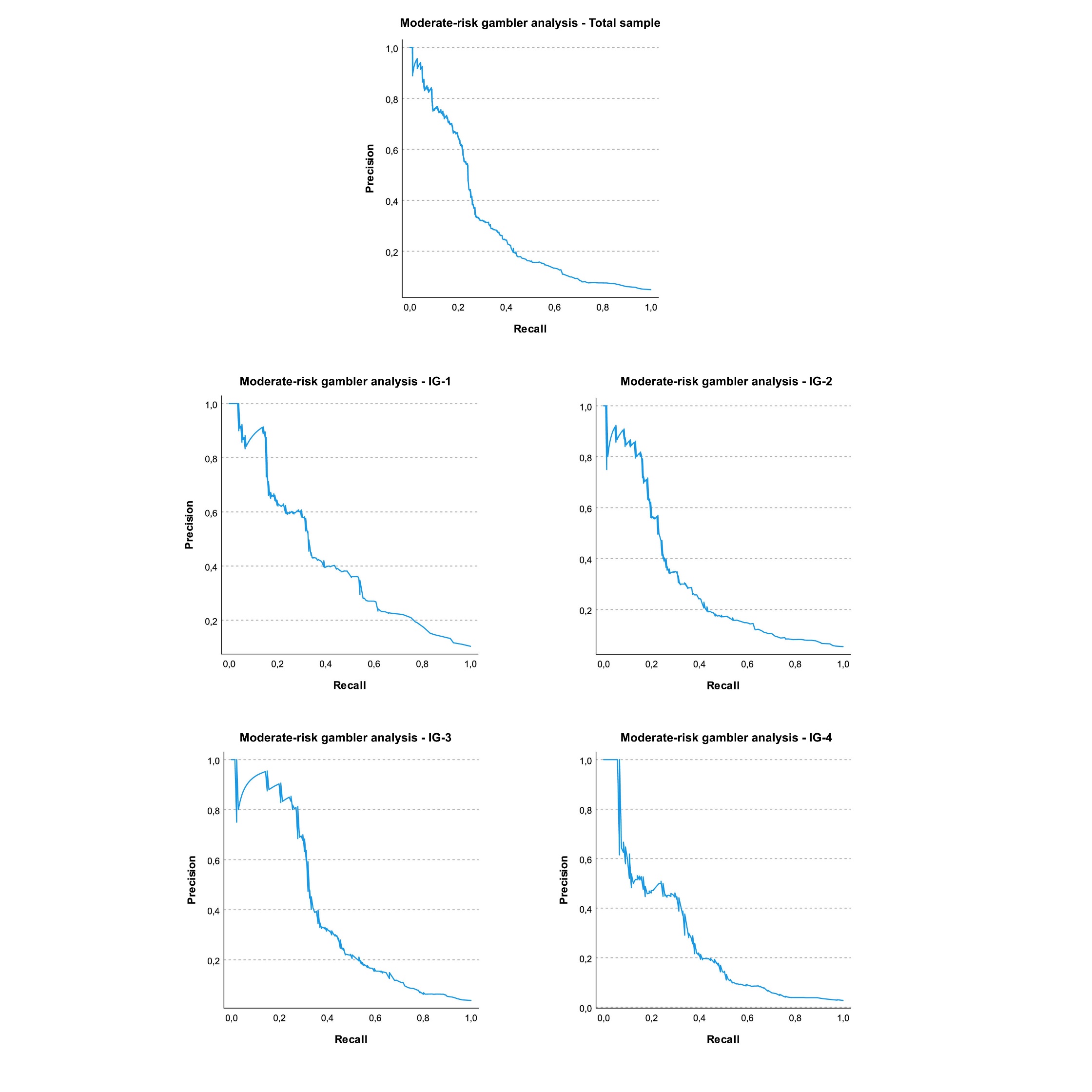


**Supplementary Figure 1.** PR curves from the moderate-risk gambler analyses for the total sample and all income groups.


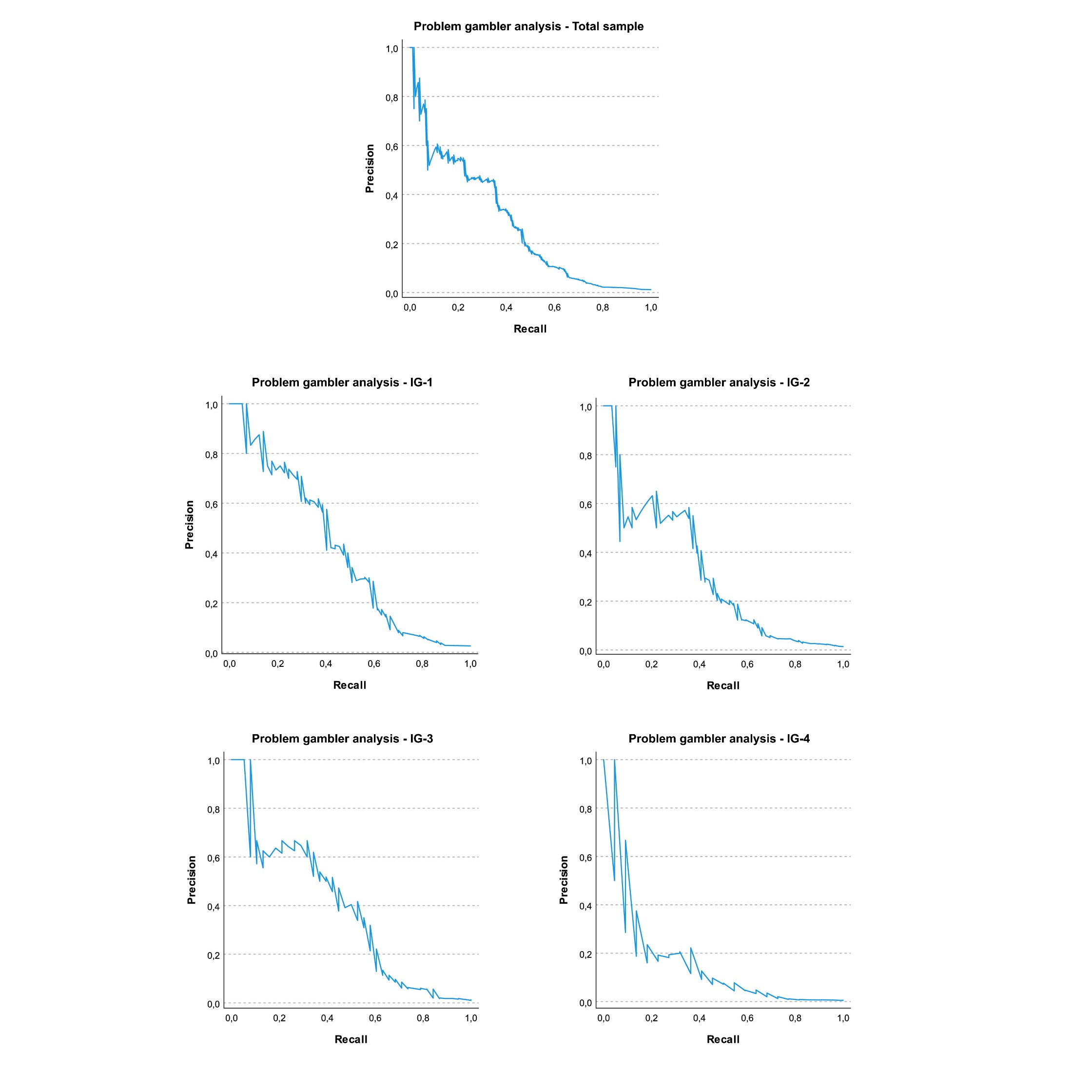


**Supplementary Figure 2.** PR curves from the problem gambler analyses for the total sample and all income groups.


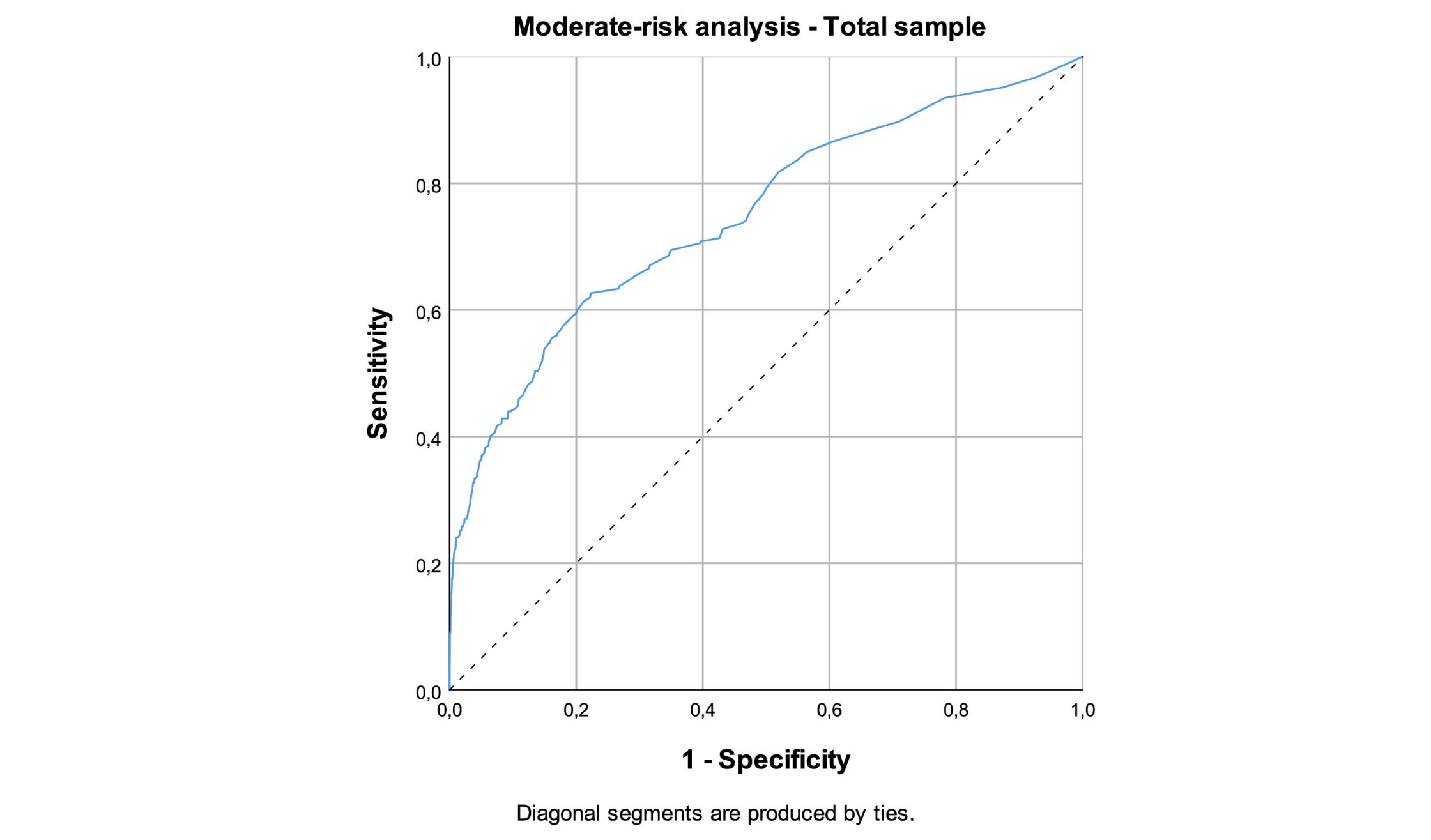


**Supplementary Figure 3.** ROC curve from the moderate-risk gambler analysis for the total sample.
